# Plasma EV-miRNAs as Potential Biomarkers of COVID-19 Vaccine Immune Response in Cancer Patients

**DOI:** 10.3390/vaccines12080848

**Published:** 2024-07-28

**Authors:** Beatriz Almeida, Tânia R. Dias, Pedro Cruz, Mário Sousa-Pimenta, Ana Luísa Teixeira, Catarina Esteves Pereira, Bruno Costa-Silva, Júlio Oliveira, Rui Medeiros, Francisca Dias

**Affiliations:** 1 Molecular Oncology and Viral Pathology Group, Research Center of IPO Porto (CI-IPOP) & RISE@CI-IPOP (Health Research Network), Portuguese Oncology Institute of Porto (IPO Porto), Porto Comprehensive Cancer Center (Porto. CCC), 4200-072 Porto, Portugal; beatriz.almeida@ipoporto.min-saude.pt (B.A.); tania.dias@ipoporto.min-saude.pt (T.R.D.); ana.luisa.teixeira@ipoporto.min-saude.pt (A.L.T.); ruimedei@ipoporto.min-saude.pt (R.M.); 2 Research Department, Portuguese League Against Cancer Northern Branch (LPCC-NRN), 4200-172 Porto, Portugal; 3 Abel Salazar Institute for the Biomedical Sciences (ICBAS), University of Porto, 4050-523 Porto, Portugal; 4 Department of Oncology, Portuguese Oncology Institute of Porto (IPO-Porto)/Porto Comprehensive Cancer Center (Porto. CCC), 4200-072 Porto, Portugal; pedro.reis.cruz@ipoporto.min-saude.pt (P.C.); julio.oliveira@ipoporto.min-saude.pt (J.O.); 5 Department of Onco-Hematology, Portuguese Oncology Institute of Porto (IPO-Porto)/Porto Comprehensive Cancer Center (Porto. CCC), 4200-072 Porto, Portugal; msousapimenta@ipoporto.min-saude.pt; 6 Systems Oncology Group, Champalimaud Research, Champalimaud Centre for the Unknown, Av. Brasília, 1400-038 Lisbon, Portugal; catarina.estevespereira@research.fchampalimaud.org (C.E.P.); bruno.costadasilva@research.fchampalimaud.org (B.C.-S.); 7 Laboratory Medicine, Clinical Pathology Department, Portuguese Oncology Institute of Porto (IPO-Porto)/Porto Comprehensive Cancer Center (Porto. CCC), 4200-072 Porto, Portugal; 8 Biomedicine Research Center (CEBIMED), Research Innovation and Development Institute (FP-I3ID), 4249-004 Porto, Portugal

**Keywords:** SARS-CoV-2, spike protein, cancer, microRNAs, EV-hsa-miR-7-5p, EV-hsa-miR-15b-5p, EV-hsa-miR-24-3p, EV-hsa-miR-145-5p, EV-hsa-miR-223-3p, cytokine storm

## Abstract

Cancer patients, prone to severe COVID-19, face immune challenges due to their disease and treatments. Identifying biomarkers, particularly extracellular vesicle (EV)-derived microRNAs (miRNAs), is vital for comprehending their response to COVID-19 vaccination. Therefore, this study aimed to investigate specific EV-miRNAs in the plasma of cancer patients under active treatment who received the COVID-19 booster vaccine. The selected miRNAs (EV-hsa-miR-7-5p, EV-hsa-miR-15b-5p, EV-hsa-miR-24-3p, EV-hsa-miR-145- 5p, and EV-hsa-miR-223-3p) are involved in regulating SARS-CoV-2 spike protein and cytokine release, making them potential biomarkers for vaccination response. The study involved 54 cancer patients. Plasma and serum samples were collected at pre-boost vaccination, and at 3 and 6 months post-boost vaccination. Anti-spike antibody levels were measured. Additionally, RNA was extracted from EVs isolated from plasma and the expression levels of miRNAs were assessed. The results showed a significantly positive antibody response after COVID-19 boost vaccination. The expression levels of EV-hsa-miR-7-5p, EV-hsa-miR-15b-5p, EV-hsa-miR-24-3p, and EV-hsa-miR-223-3p increased significantly after 6 months of COVID-19 booster vaccination. Interestingly, an increased expression of certain EV-hsa-miRNAs was positively correlated. Bioinformatic analysis revealed that these correlated miRNAs play a critical role in regulating the targets present in antiviral responses and cytokine production. These findings suggest that EV-hsa-miR-15b-5p, EV-hsa-miR-24-3p, and EV-hsa-miR-223-3p may be crucial in immune response induced by mRNA vaccines.

## 1. Introduction

Severe acute respiratory syndrome virus-2 (SARS-CoV-2), a novel *Betacoronavirus* and a typically single-stranded positive-sense RNA virus (+ssRNA) with approximately 79% sequence homology with SARS-CoV and 50% homology with MERS-CoV, was the causative agent of the pandemic that started at the end of 2019, with the first case of coronavirus disease 2019 (COVID-19) described in Wuhan, China [1,2,3]. Since this case, SARS-CoV-2 viral infection has spread rapidly within a short period of time. In fact, COVID-19 was declared by World Health Organization (WHO) on 11 March 2020 as a global pandemic responsible for more than six million deaths worldwide, demonstrating that this illness still has an impact on public health. To reduce the high number of cases that COVID-19 has caused and manage the pandemic, there was a worldwide effort to develop vaccines and therapy options, which have led to the emergence of mRNA vaccines against COVID-19 [4,5,6].

mRNA vaccines have been administered using various formats, including encapsulation by delivery carriers like lipid nanoparticles (LNPs), polymers and peptides, as well as free mRNA in solution and ex vivo delivery through dendritic cells (DCs) [7,8,9]. Particularly, LNPs serve as an effective delivery system for mRNA vaccines, specifically targeting DCs [10]. As a result, the delivery of mRNA vaccines using lipid nanoparticles stimulates both cellular and humoral immune responses, promoting better vaccine efficacy [11,12]. Nonetheless, lipid nanoparticles used for vaccine delivery possess similarities to the endogenous lipid nanoparticles found in human organisms [13,14,15]. This characteristic makes them favorable carriers for mRNA vaccination, since the immune system recognizes the lipid nanoparticle with mRNA of the viral target, without being identified as foreign entities that need to be eliminated [16,17]. mRNA-1273 and BNT162b2 vaccines trigger the immune system to produce neutralizing antibodies (NAbs) against SARS-CoV-2 spike proteins, proposed as the major correlate of protection, promoting robust germinal center responses in humans, and resulting in memory B cells that are specific for both the full-length SARS-CoV-2 spike protein and the spike-receptor-binding domain (RBD) [18,19,20]. Moreover, mRNA vaccination has also been shown to generate spike-specific memory CD4+ and CD8+ T cell responses [21,22]. There are other benefits of manipulating mRNA to allow for host cells to produce viral antigenic protein fragments. One key benefit is that producing mRNA is easier compared to the traditional method of whole viral inactivation [23]. Additionally, the transcription reaction is easy to perform, produces a high yield, and can be scaled up efficiently; also, mRNA vaccines enable the synthesis of antigen proteins in situ, eliminating the need for protein purification and long-term stabilization, which are challenging for antigen transportation, and the storage of mRNA may be easier than protein-based vaccines, since RNA, if protected properly against ribonucleases (RNases), is less prone to degradation compared to proteins [24]. Establishing immunity against SARS-CoV-2 has become a central focus of current research efforts. Findings from the human trials of the Pfizer/BioNTech mRNA vaccine demonstrated 95% maximal protection within seven or more days after the second vaccine dose, including against several circulating variants of concern [25].

However, it is known that there are some individuals more prone to SARS-CoV-2 infection due to immunosuppression (either caused by cancer or other immune disorders), and this fact also applies to their capacity of vaccination response [26,27]. Since cancer patients are not included in vaccination clinical trials, there is still considerable uncertainty in terms of the efficacy of the SARS-CoV-2 vaccines that are available, as well as the extent of humoral and cellular immune responses and the impact of the related side effects [28]. In addition to an increased susceptibility to SARS-CoV-2 infection, the immunosuppressive state of cancer patients makes them more prone to vaccination failure [29,30]. The investigation of how the mRNA of spike protein interacts with host molecular regulatory systems is crucial to better understand the impact of the mRNA vaccines in the immune system of cancer patients [31]. To evaluate and provide more details about how COVID-19 vaccination affects the cancer patients, it is necessary to have measurable biomolecules that can indicate the impact on the immune system of these individuals more precisely.

Therefore, the miRNA content of EVs can be a promising biomarker for the evaluation of vaccination responses in immunocompromised people, such as cancer patients [32,33]. EV-derived miRNAs can be useful biomarkers since they have the advantage of being obtained through minimally invasive methods, such as liquid biopsies [32,34,35]. In fact, miRNAs transported in EVs exhibit attractive advantages in the biomarker discovery field, since the lipid bilayer of EVs can protect them from RNase degradation while circulating in the body, and they are also easy to quantify through well-established molecular biology techniques, such as real-time PCR [36,37]. With the establishment of the important role of microRNAs in regulating target mRNAs and, consequently, protein expression, Wang and colleagues demonstrated that hsa-miR-7-5p, hsa-miR-15b-5p, hsa-miR-24-3p, hsa-miR-145-5p, and hsa-miR-223-3p were able to directly target the S protein mRNA and inhibit SARS-CoV-2 replication [38]. In addition to that, a review article from our research group has described that these microRNAs are deregulated in most cancer types and are involved in immune response processes, such as the cytokine storm [39]. Therefore, it is possible that cancer patients with an aberrant expression of these miRNAs that are also able to regulate the expression of spike protein will be more prone to vaccination failure. However, this miRNA profile has not yet been validated in cancer patients in the context of SARS-CoV-2 susceptibility or vaccination response. Taking all this into account, EV-hsa-miRNAs capable of interfering with the spike protein of SARS-CoV-2 seem to be a promising approach in the search for biomarkers capable of stratifying patients according to the degree of their immune response to vaccination, and provide more insight about the response process in cancer patients. Therefore, in this study, we assessed the long-term humoral immune response, demonstrating that most of the recruited patients had positive antibody levels after SARS-CoV-2 vaccination. Additionally, we tried to identify an EV-hsa-miRNAs profile in cancer patients that could be used to stratify them according to their humoral response and provide more insight on the potential signaling pathways involved in that response. Based on our results, we can hypothesize that the elevated plasma levels of some EV-hsa-miRNAs could be a consequence of COVID-19 vaccination and could be associated with specific immunological agents related to cellular immunity. The establishment of vaccination response biomarkers can ultimately lead to a more specific management of this high-risk sub-population, with an improvement in personalized medicine approaches.

## 2. Materials and Methods

### 2.1. Study Population and Sample Storage

The analysis of the EV-derived miRNA profile was conducted through a hospital-based study involving a total of 54 cancer patients from the north of Portugal, diagnosed and followed at IPO-Porto (Table 1). The project was approved by the IPO-Porto ethics committee (Reference: CES IPO:33/022), and the individuals enrolled in the study signed their written informed consent, in agreement with the principles of the Helsinki Declaration. The inclusion criteria were as follows: cancer patients with age above 18 years, under active treatment, and eligible for a booster dose of COVID-19 vaccination. Blood samples were collected from all patients (2 EDTA tubes + 1 serum separator tube) at three timepoints: before COVID-19 vaccine booster dose, 3 months after booster dose, and 6 months after booster dose. The samples were centrifuged at 3000× *g* for 5 min to separate the blood fractions. The plasma and serum samples were stored at −80 °C, and the whole blood samples were stored at −20 °C until further processing. 

### 2.2. Quantification of Anti-SARS-CoV-2 IgG Antibody Levels in Immunoessay ELISA

To detect specific anti-SARS-CoV-2 Spike IgG antibodies, 200 μL of serum samples from each timepoint were examined using the Atellica^®^ IM SARS-CoV-2 IgG (sCOVG) immunoassay (Siemens Healthineers^TM^ Erlangen, Deutschland). The procedure followed the manufacturer’s protocol.

### 2.3. Plasma Purification and Extracellular Vesicle Isolation

The plasma samples were subjected to three additional rounds of centrifugation, with increasing speeds of 400× *g*, 2100× *g*, and 10,000× *g*, each lasting 15 min. This process was performed to obtain platelet-free plasma (PFP). After centrifugation, the supernatant was collected and filtered through a 0.22 μM filter (GE Healthcare Whatman^TM^ Chicago, IL, USA) to remove any remaining impurities. Next, 200 μL of PFP was treated with proteinase K at 37 °C for 10 min, followed by the addition of Total Exosome Isolation (TEI) reagent (Invitrogen™ Waltham, MA, USA). The solution was then incubated for 30 min at 4 °C. The EVs were precipitated by 5 min of centrifugation at 10,000× *g* at room temperature. The resulting pellet, which contained the pre-enriched EVs, was resuspended in 100 μL of filtered PBS and stored at −20 °C until further analysis.

### 2.4. Extracellular Vesicle NTA Analysis

Nanoparticle Tracking Analysis (NTA) was used to measure the relative concentration and size distribution of the EVs. Measurements were obtained using the NS300 Nanoparticle Tracking Analysis (NTA) system (NanoSight—Malvem Panalytical, Malvern, UK). Samples were pre-diluted at 1:100 in filtered PBS to achieve a concentration within the range for optimal NTA analysis. After a pre-washing step with filtered PBS, the samples were dispensed into the sample chamber at a constant flow rate using the Malvern NanoSight syringe pump system with a pump speed of 15. The capture settings were as follows: sCMOS camera, red laser, slider shutter of 1200, slider gain of 500, and a temperature of 24.5 °C. All samples presented more than 20 particles per frame, and a total of 749 frames were acquired per sample. Video acquisitions were performed using a camera level of 15 (NTA 3.0 levels), and five to nine videos of 30 s were recorded per sample. Analysis of particle concentration per mL and size distribution was performed with NTA 3.4 Build 3.4.003 software. For this characterization, we randomly selected 3 patients and analyzed all their timepoint samples (pre-boost, 3 months post-boost, and 6 months post-boost), making a total of 9 samples analyzed.

### 2.5. Transmission Electron Microscopy Characterization of EVs

To validate the presence of EVs and evaluate their morphology, the EV samples were analysed by Transmission Electron Microscopy (TEM) at i3S Histology and Electron Microscopy facility using a Jeol JEM 1400 transmission electronic microscope (Tokyo, Japan) and images were acquired at different ampliations.

### 2.6. EV-CD63 Quantification 

To further confirm the existence of EVs in our samples, an EV-CD63 quantification using the Human CD63 ELISA Kit (Thermofisher^TM^ Waltham, MA, USA) was carried out. CD63 (LAMP-3, lysosome-associated membrane protein-3) is a glycoprotein extensively expressed in EVs surfaces, and for that reason, widely used as an EV biomarker. Therefore, based on a solid-phase sandwich Enzyme-Linked Immunosorbent Assay (ELISA), EV-CD63 was detected and quantified using a sample volume of 100 μL from 4 patients at the 3 timepoints studied, making a total of 12 samples analyzed. 

### 2.7. RNA Extraction and cDNA Synthesis

After EV isolation, the microRNA extraction was performed using the MagMAX^TM^ mirVana^TM^(Thermofisher^TM^ Waltham, MA, USA) Total RNA Isolation Kit in the KingFisher Flex extractor (ThermoFisher^TM^ Waltham, MA, USA). RNA concentration and purity was assessed through a NanoDrop Lite^TM^ spectrophotometer (ThermoFisher^TM^ Waltham, MA, USA), and the total RNA values were used as templates for cDNA synthesis. For quantification of microRNAs, cDNA synthesis was performed using a Taqman^®^ MicroRNA Reverse Transcription kit (Applied Biosystems^TM^ Waltham, MA, USA) and sequence-specific stem–loop primers for miR-7-5p, miR-15b-5p, miR-24-3p, miR-145-5p, miR-223-3p, miR-21, and let-7a-5p. After protocol optimization, the thermal conditions were as follows: 16 °C for 30 min, followed by 42 °C for 60 min and 85 °C for 10 min.

### 2.8. Quantification of the Selected EV-hsa-miRNAs Profile by Real-Time Quantitative Polymerase Chain Reaction (RT-qPCR)

MicroRNA expression levels were analyzed by quantitative real-time PCR. The reactions were carried out in a StepOnePlus^TM^ (ThermoFisher^TM^ Waltham, MA, USA) qPCR Real-Time PCR machine, in a volume of 10 μL containing 1X TaqMan^TM^(ThermoFisher^TM^ Waltham, MA, USA) Fast Advanced Master Mix (Applied Biosystems™), with 1× TaqMan^®^ miRNA Expression Assays probes (miR-7-5p: 005723_mat; miR-15b-5p: 000390; miR-24-3p: 000402; miR-145-5p: 477916_mir; miR-223-3p: 002295; and miR-21: 000397—Applied Biosystems^TM^), and 4 μL of cDNA. For microRNA expression normalization, two housekeeping controls were used: GAPDH (Hs99999905_m1—Applied Biosystems^TM^) and let-7a-5p (000377—Applied Biosystems^TM^). These housekeeping genes were chosen since they are reported as typical EV cargo. The amplification conditions were as follows: holding stage 95 °C for 20 seconds, followed by 45 cycles of 95 °C for 1 second and 60 °C for 20 seconds. Two technical replicates were made for each sample and negative controls were also included in each run. Data analysis was conducted using StepOne^TM^ Sofware version 2.2 (Applied Biosystems^TM^) with the same baseline and threshold set for each plate, to generate cycle threshold (Ct) values for all the mRNAs in each sample.

### 2.9. Data Analysis

Since the quantification of microRNA expression was carried out using TaqMan miRNA expression assays (Applied Biosystems^TM^) through quantitative real-time PCR, it was possible to perform a comparative quantification using the Livak–Schmittgen method. The statistical analysis of the results was conducted using SPSS software for Windows (version 29.0) and GraphPad Prism (version 8.0). After confirming normality parameters through Shapiro–Wilk and Kolmogorov–Smirnov tests, group differences were evaluated using either an unpaired *t*-test or Mann–Whitney *U* test. The let-7a-5p endogenous control exhibited stable behavior, so it was used to normalize the EV-microRNA levels. The EV-derived miRNA levels (low versus high) were defined using the mean value of the ΔCt. The Kaplan–Meier method and Log Rank test were used to establish the association of the EV-derived miRNA levels (low and high) and clinical variables to the patient’s overall survival. Furthermore, Spearman correlation was conducted to study the relationship between EV-hsa-miRNA expression patterns, anti-Spike IgG levels, and the EV-hsa-miRNAs. To identify the common targets of the EV-hsa-miRNAs that presented a robust Spearman correlation coefficient, an in silico analysis was conducted through miRTargetLink 2.0 software.

## 3. Results

### 3.1. Humoral Response to COVID-19 Vaccination

**Figure 1:**
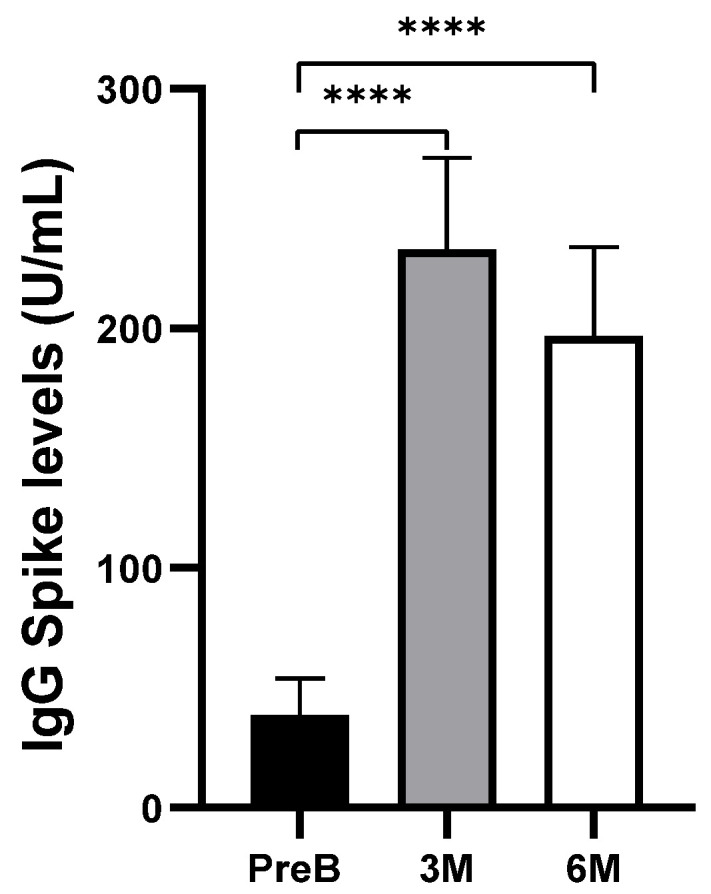
Anti-spike IgG response of cohort cancer patients in pre-boost (PreB) vaccination, 3 months after (3 M) boost vaccination, and 6 months (6 M) after boost vaccination. The mean ± SEM of each timepoint was 38.50 ± 15.27 U/mL, 232.9 ± 38.30 U/mL, and 196.8 ± 36.99 U/mL, respectively. Differences were tested by Mann–Whitney *U* test. **** *p* < 0.0001. Obtained with GraphPad Prism (version 8.0).

Anti-SARS-CoV-2 Spike IgG antibodies from serum samples were analyzed through ELISA immunoassay (Figure 1). In this analysis, levels of IgG antibodies against S1 of spike protein in cancer patients significantly increased after 3 months of boost-vaccination (*p* < 0.0001) and remained high after 6 months of boost vaccination (*p* < 0.0001). 

However, it is important to note that not all the patients presented a humoral response before and/or after vaccination boost. In fact, we observed that most of the non-responders were the patients with hematologic tumors (Table 2).

### 3.2. Extracellular Vesicle Characterization

**Figure 2:**
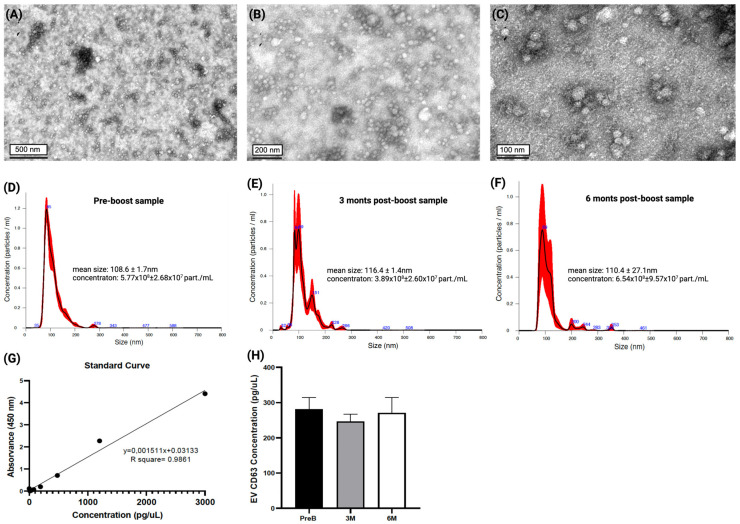
Extracellular vesicle characterization. (**A**–**C**) Transmission electron microscopy (TEM) images of EVs from purified platelet-free plasma (PFP) of cancer patient at (**A**) 25,000 ×(0.5 um scale), (**B**) 50,000× (200 nm scale), and (**C**) 100,000× (100 nm scale). (**D**–**F**) Nanoparticle tracking analysis (NTA) of EVs derived from plasma samples of cancer patient at 3 defined timepoints. The red error bars indicate ± 1 standard error of the mean. (**G**) Standard curve of the ELISA assay for EV-CD63 concentration assessment of the 12 samples analyzed. (**H**) EV-CD63 concentration of plasma samples from 4 cancer patients in the three timepoints analyzed.

The EVs were characterized according to size, morphology, and purity. The TEM images (Figure 2A–C) show the variability in sizes and morphology present in EVs isolated from a patient’s PFP at different scales. The TEM images displayed rounded structures with a predominantly white appearance and size range in nanometers. This morphology and size are indicative of the EVs. 

We also did NTA analysis on random samples of the three timepoints studied (Pre-boost, 3 months post-boost, and 6 months post boost) to determine if there were considerable changes in size and concentration among them. Figure 2D–F show that the size and concentration of particles detected at the defined timepoints remain stable and that the size distribution is around 100 nm, which is consistent with the size of EVs. Furthermore, CD63 was also successfully detected in the EV samples using a Human CD63 ELISA assay (Figure 2G,H). Since CD63 is highly enriched in EV membranes, we analyzed the samples of four patients at the three timepoints and successfully detected anti-CD63 marked EVs in the 12 samples analyzed. Additionally, these concentrations showed no statistically significant differences across the three timepoints.

### 3.3. EV-hsa-miRNA Profile Levels in Cancer Patients’ Plasma Samples and Their Relationship with Humoral Response

**Figure 3:**
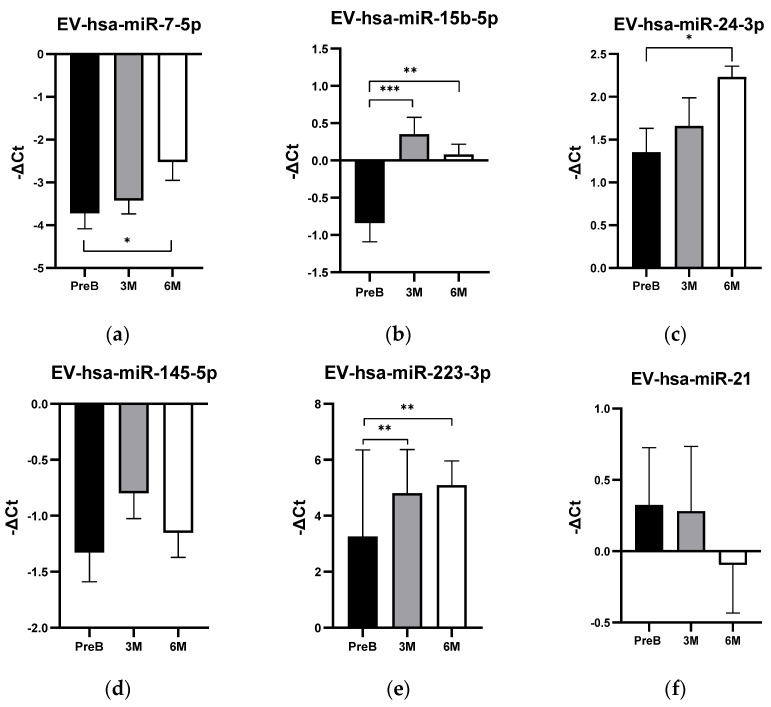
Plasma EV-derived miRNA expression (−∆Ct) in pre-boost (PreB) vaccination, 3 months (3 M) post-boost vaccination, and 6 months (6 M) post-boost vaccination samples of cancer patients from cohort population. (**a**) EV-hsa-miR-7-5p plasmatic EV-expression with mean ± SEM of PreB (N = 54): −3.72 ± 0.36; 3 M (N = 50): −3.42 ± 0.31; 6 M (N = 40): −2.53 ± 0.43; (**b**) EV-hsa-miR-15b-5p plasmatic EV-expression with mean ± SEM of PreB (N = 54): −0.84 ± 0.25; 3 M (N = 50): 0.35 ± 0.23; 6 M (N = 40): 0.079 ± 0.14; (**c**) EV-hsa-miR-24-3p plasmatic expression with mean ± SEM of PreB (N = 54): 1.35 ± 0.28; 3 M (N = 50): 1.66 ± 0.33; 6 M (N = 40): 2.23 ± 0.13; (**d**) EV-hsa-miR-145-5p plasmatic expression with mean ± SEM of PreB (N = 54): −1.33 ± 0.26; 3 M (N = 50): −0.80 ± 0.23; 6 M (N = 40): −1.15 ± 0.22; (**e**) EV-hsa-miR-223-3p plasmatic expression with mean ± SEM of PreB (N = 54): 3.26 ± 0.42; 3 M (N = 50): 4.81 ± 0.22; 6 M (N = 40): 5.09 ± 0.14; (**f**) EV-hsa-miR-21 plasmatic expression with mean ± SEM of PreB (N = 30): 0.3237 ± 0.4020; 3 M (N = 30): 0.2807 ± 0.4538; 6 M (N = 29): −0.09505 ± 0.3375. Differences were tested by unpaired *t*-test or Mann–Whitney *U* test. * *p* < 0.05, ** *p* < 0.01, *** *p* < 0.001. Obtained with GraphPad Prism (version 8.0).

The expression levels of the selected five EV-hsa-miRNAs (EV-hsa-miR-7-5p, EV-hsa-miR-15b-5p, EV-hsa-miR-24-3p, EV-hsa-miR-145-5p, and EV-hsa-miR-223-3p) in plasma samples of the studied population are represented in Figure 3. Since this is still an ongoing study that is still collecting samples and follow-up data, it was not possible to analyze all 54 patients in all defined timepoints. Therefore, 54 plasma samples were analyzed in pre-boost vaccination, 50 plasma samples were analyzed 3 months post-boost vaccination, and 40 plasma samples were analyzed 6 months post-boost vaccination.

Overall, most of the selected EV-hsa-miRNAs demonstrated an increase in expression levels after the vaccination boost. At the 3-month post-boost timepoint, we observed an increase in the expression levels of the EV-hsa-miR-15b-5p (*p*-value = 0.0007) and EV-hsa-miR-223-3p (*p*-value = 0.0019) (Figure 3b,e, respectively). 

At the 6-month post-boost timepoint, EV-hsa-miR-15b-5p and EV-hsa-miR-223-3p also showed increased expression levels compared to the pre-boost state (hsa-miR-15b-5p: *p*-value = 0.0049; hsa-miR-223-3p: *p*-value = 0.0005). Additionally, there was an increase in EV-hsa-miR-7-5p (*p*-value = 0.0309) and EV-hsa-miR-24-3p (*p*-value = 0.0118) (Figure 3a,c, respectively). It is important to note that EV-hsa-miR-7-5p was not detected in all the samples analyzed. There were no statistically significant differences in EV-hsa-miR-145-5p expression levels at 3 and 6 months post-boost, respectively (*p*-value = 0.2381; *p*-value = 0.8880) (Figure 3d). 

To further assess the potential relationship between the selected miRNA profile and COVID-19 vaccination, we evaluated another EV-miRNA, EV-hsa-miR-21, which has no experimentally established relation to the spike protein, in 30 cancer patients of our cohort. The expression levels of EV-hsa-miR-21 did not show significant differences when comparing pre-boost vaccination to 3 months post-boost (*p*-value = 0.9437), pre-boost to 6 months post-boost (*p*-value = 0.4298), and 3 months to 6 months post-boost (*p*-value = 0.5113) (Figure 3f). Given that this microRNA is frequently studied for its oncogenic role in the PI3K-Akt pathway across various cancers, it can be concluded that, within our heterogeneous cohort, the expression levels of this microRNA do not change following COVID-19 vaccination.

The association between the humoral immunity and the EV-hsa-miRNAs through Spearman correlation determined no significant correlation between the levels of anti-SARS-CoV-2 spike IgG in cancer patients with the EV-hsa-miRNAs profile expression levels at 3 months or 6 months post-vaccination boost (Table 3 and Table 4). These results suggest that the elevation of expression levels of EV-hsa-miRNAs following the booster dose does not correlate with the humoral response to COVID-19 vaccination in cancer patients.

### 3.4. Overall Survival of Cancer Patients According to Clinical Variables and Low or High Expression of the EV-hsa-miRNA Profile

To investigate whether the selected EV-hsa-miRNA profile was associated with clinical variable overall survival (OS), the association of high and low EV-hsa-miRNAs in pre-boost vaccination samples with OS was analyzed, considering the following clinical variables: gender, tumor type, COVID-19 vaccine type, COVID-19 infection, smoking status, dyslipidemia, hypertension, and diabetes.

**Figure 4:**
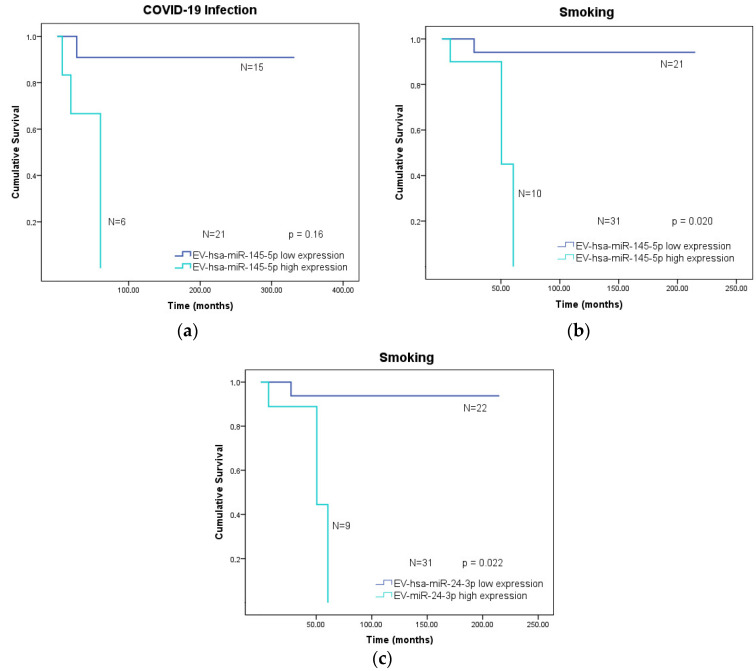
Overall survival analysis with Kaplan–Meier curves of cancer patients with low or high expression levels of specific EV-hsa-miRNAs (N = 54). (**a**) Overall survival of cancer patients who had COVID-19 infection and expression levels of EV-hsa-miR-145-5p (*p*-value = 0.016). (**b**) Overall survival of smoker cancer patients and expression levels of EV-hsa-miR-145-5p (*p*-value = 0.020). (**c**) Overall survival of smoker cancer patients and expression levels of EV-hsa-miR-24-3p (*p*-value = 0.022). Obtained in SPSS software (Version 29.0).

Statistical differences were found regarding COVID-19 infection and smoking habits (Figure 4). High expression levels of EV-hsa-miR-145-5p in the group of patients who had COVID-19 were associated with poorer overall survival in these cancer patients (*p*-value = 0.016; Figure 4a). Moreover, high expression of this EV-hsa-miRNA in smoker cancer patients also induced a worse overall survival (*p*-value = 0.020; Figure 4b). Similarly, high expression levels of EV-hsa-miR-24-3p in smoker cancer patients led to a worse overall survival outcome (*p*-value = 0.022; Figure 4c). 

### 3.5. Correlation between EV-hsa-miRNAs

**Figure 5:**
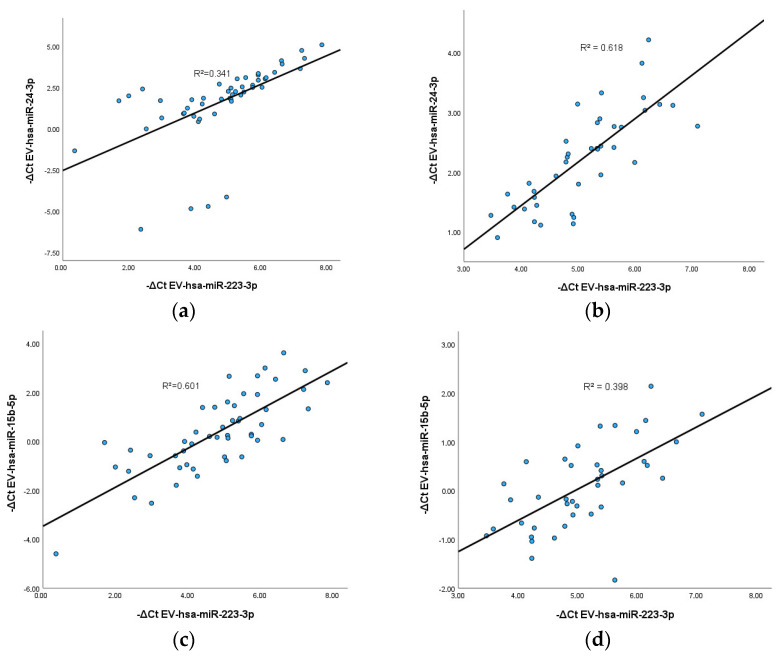
Correlation plots. (**a**) Correlation plot between EV-hsa-miR-223-3p and EV-hsa-miR-24-3p at 3 months after boost vaccination (Linear R^2^ = 0.341; Spearman rho = 0.843; *p* < 0.001). (**b**) Correlation plot between EV-hsa-miR-223-3p and EV-hsa-miR-24-3p at 6 months after boost vaccination (Linear R^2^ = 0.618; Spearman rho = 0.792; *p* < 0.001). (**c**) Correlation plot between EV-hsa-miR-223-3p and EV-hsa-miR-15b-5p at 3 months after boost vaccination (Linear R^2^ = 0.601; Spearman rho = 0.768; *p* < 0.001). (**d**) Correlation plot between EV-hsa-miR-223-3p and EV-hsa-miR15b-5p at 6 months after boost vaccination (Linear R^2^ = 0.398; Spearman rho = 0.639; *p* < 0.001). Obtained with SPSS software (version 29.0).

Since the results indicate a general increase in EV-hsa-miRNAs after COVID-19 boost vaccination, it was investigated whether there was a correlation between these EV-hsa-miRNAs at the analyzed timepoints. The statistically significant results, supported by the curve fit of correlation, are illustrated in Figure 5. The results indicate a positive correlation between EV-hsa-miR-223-3p and two other EV-hsa-miRNAs: EV-hsa-miR-24-3p and EV-hsa-miR-15b-5p. The Spearman correlation analysis further reveals a positive correlation between EV-hsa-miR-223-3p and EV-hsa-miR-24-3p at 3 and 6 months post-boost vaccination (Figure 5a,b), with a correlation coefficient of 0.843 and 0.792, respectively. Similarly, EV-hsa-miR-223-3p demonstrates a positive correlation with EV-hsa-miR-15b-5p (Figure 5c,d), exhibiting a correlation coefficient in 0.768 at 3 months after boost vaccination and a slight correlation coefficient of 0.639 at 6 months after boost vaccination. These results suggest that these EV-hsa-miRNAs may be stimulated to play a synergistic role in molecular pathways associated with response to COVID-19 vaccination, with a positive correlation observed in their expression levels over time. However, since the correlation value between the miRNAs is positive, R^2^ should be close to 1 for high correlation, so these results should be interpreted carefully.

### 3.6. Common Targets of EV-hsa-miR-223-3p with EV-hsa-miR-24-3p and with EV-hsa-miR-15b-5p

**Figure 6:**
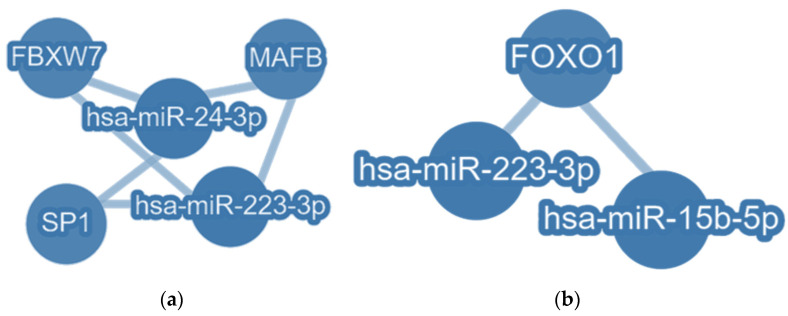
In silico approach of EV-hsa-miRNAs with positive Spearman correlation. (**a**) Common strongly validated targets of hsa-miR-223-3p and hsa-miR-24-3p. (**b**) Common strongly validated targets of EV-hsa-miR-223-3p and EV-hsa-miR-15b-5p. Obtained with miRTargetLink 2.0 software.

Since it was observed that EV-hsa-miR-24-3p, EV-hsa-miR-15b-5p, and EV-hsa-miR-223-3p were statistically elevated after COVID-19 vaccination in cancer patients and there is a significant positive correlation between them, studying the genes commonly targeted by these EV-hsa-miRNAs was essential to understand the biological effects of their overexpression. Therefore, an in silico analysis presented in Figure 6 highlights shared experimentally validated targets between hsa-miR-223-3p and hsa-miR-24-3p, as well as between hsa-miR-223-3p and hsa-miR-15b-5p. The findings demonstrate that *FBXW7*, *MAFB*, and *Sp1* genes are commonly targeted by both hsa-miR-223-3p and hsa-miR-24-3p. Additionally, *FoxO1* emerges as the exclusive strongly validated common target between hsa-miR-223-3p and hsa-miR-15b-5p. 

## 4. Discussion

Cancer patients, at high risk for severe COVID-19, are prioritized for vaccination. Monitoring anti-SARS-CoV-2 spike IgG levels is crucial for assessing long-term humoral immune response, as the spike protein and its mRNA are pivotal in vaccine formulation and immune protection. The data showed that most of the patients had positive antibody levels at 3 and 6 months after boost vaccination. These results align with previous studies that concluded that patients with cancer developed anti-SARS-CoV-2 spike IgG above the threshold level after partial and complete immunization. Nevertheless, a noticeable decline in antibodies was observed between the 3- and 6-month post-boost vaccination timepoints, indicating a decrease in the humoral immune response against SARS-CoV-2 over time. Research in this field has demonstrated that, in the general population, antibodies generated by COVID-19 vaccines can diminish in as little as 3 to 10 weeks after the second dose [40,41]. This result reveals the importance of regularly stimulating the immune system to maintain robust defenses against SARS-CoV-2 infection. Furthermore, studies distinguish between hematological and solid cancer patients, demonstrating that hematological cancer patients have lower serum antibody levels compared to cancer patients with solid tumors [42,43]. Despite the limited number of cancer patients with hematological tumors in our study, we observed an absence of humoral immune response in hematologic cancer patients after boost vaccination, compared to cancer patients with solid tumors, with most of the patients presenting a negative IgG spike response. This confirms the influence of the immunosuppressive state in the immune response of these individuals [44]. However, it is important to note that, due to the small size of the group with hematological malignancies of our cohort, no further statistical analysis was carried out. Additionally, it has been reported that cancer patients undergoing various anti-cancer therapies elicit distinct humoral responses [45,46]. In fact, Janzic and co-workers observed that, at 3 months after COVID-19 vaccination, there was a decline in anti-SARS-CoV-2 S1 IgG levels in patients receiving chemotherapy and immune checkpoint inhibitors, but not targeted therapy, when compared to healthy donors [46]. Future analysis should include more hematological malignancy patients and diverse treatment subgroups to explore the influence of malignancy type and treatment on vaccination-induced humoral responses.

Regarding EV-miRNA expression levels, the results reveal that at 6 months after boost vaccination, there is an increase in EV-hsa-miR-7-5p, EV-hsa-miR-15b-5p, EV-hsa-miR-24-3p, and EV-hsa-miR-223-3p levels. Since these EV-hsa-miRNAs can inhibit the viral translation after the attachment of miRNAs to 3′-UTR of the viral mRNA encoding spike protein, it can be hypothesized that the increase in these EV-hsa-miRNAs may attenuate induced inflammation caused by COVID-19 boost vaccination, suppressing the expression of transcription and translation machinery involved in virus replication and translation. In fact, studies demonstrate that EVs or miRNAs modulate host factors to normalize immunopathogenesis, having an antiviral effect in host cells by interacting directly with viral RNA to prevent its replication [47]. Other studies, such as the research carried out by Mishra and Banerjea, proposed that the spike protein can modify the host exosome cargo, which gets transported to distant uninfected tissues and organs and can initiate a catastrophic immune cascade [48]. Moreover, circulating exosomes with SARS-CoV-2 spike protein can also have the potential to induce an immune response because of mRNA-based vaccine administration in cancer patients [49]. Thus, the increase in EV-hsa-miR-7-5p, EV-hsa-miR-15b-5p, EV-hsa-miR-24-3p, and EV-hsa-miR-223-3p after vaccine boost can be a possible response of the organism to the exacerbation of a viral mRNA element, this being the organism’s immune reaction to the intense presence of a viral protein. However, no significant correlation was found between the antibodies and EV-hsa-miRNA levels. Since we observed a positive humoral immune response in cancer patients via anti-SARS-CoV-2 spike IgG antibodies at 3 and 6 months post-boost vaccination, it can be hypothesized that the increase in EV-hsa-miRNAs observed at the same timepoint does not significantly influence the humoral immune response. This opens an intriguing path to investigate the potential influence of EV-hsa-miRNAs on cell-mediated immunity in future projects.

Regarding the overall survival analysis, we observed that cancer patients who had previously contracted SARS-CoV-2 and exhibited high expression levels of EV-hsa-miR-145-5p were more likely to experience a poorer outcome compared to those with low expression levels of this EV-hsa-miRNA. In fact, the implications of an elevated expression of EV-miR-145-5p and the occurrence of severe COVID-19 symptoms have been reported. Guiot and colleagues demonstrated that EV-miR-145-5p is markedly upregulated in the blood of both COVID-19 and idiopathic pulmonary fibrosis patients [50]. The upregulation of this pro-fibrotic microRNA can potentially induce lung fibrosis in COVID-19 patients, exacerbating the severity of the disease. Therefore, it is plausible to hypothesize that this EV-hsa-miRNA may significantly impact the manifestation of severe symptoms in COVID-19 patients with cancer, with those having elevated levels of this EV-hsa-miRNA more likely to face a more challenging prognosis in terms of overall survival. In a separate set of results, it was additionally noted that elevated levels of EV-hsa-miR-24-3p and EV-hsa-miR-145-5p are associated with a lower overall survival of cancer patients who smoke. Once more, the interplay between cancer-induced immunosuppression and a substantial risk factor like smoking aggravates the difficulties faced by these patients. This, in turn, appears to contribute to a more adverse survival scenario for individuals with heightened expression of EV-hsa-miR-24-3p and EV-hsa-miR-145-5p. Concerning the role of these EV-hsa-miRNAs in smoking-related complications, there is a lack of information regarding the specific molecular mechanisms of these microRNAs in smoker cancer patients, leading to a worse overall survival. However, studies demonstrate that both miR-145-5p and miR-24-3p were associated with the pathophysiology of COPD related to cigarette smoke in non-cancer patients [51,52]. Based on these data, we can hypothesize that smoking cancer patients with high levels of EV-miR-145-5p and miR-24-3p may be more prone to inflammatory states in the lung, which can influence their survival.

During the EV-miRNA analysis, it was interesting to note a positive correlation between the expression levels of EV-hsa-miR-223-3p and those of both EV-hsa-miR-24-3p and EV-hsa-miR-15b-5p. Notably, EV-hsa-miR-223-3p and EV-hsa-miR-24-3p were identified as potential serum biomarkers for pneumonia, a severe symptom in COVID-19. In the context of pneumonia, the expression levels of these miRNAs are characterized by low levels of hsa-miR-24 and high levels of hsa-miR-223 [53]. Furthermore, other studies evidence that both EV-hsa-miR-223-3p and EV-hsa-miR-15b-5p are recognized as biomarkers in asthma. According to research conducted by Hirai et al., circulating miR-15b-5p holds promise as a biomarker for identifying asthma-COPD overlap patients [54,55]. On the other hand, the main function of miR-223-3p is in the pathophysiology of asthma sustains in pro-inflammatory activity of human bronchial epithelial cells [56,57]. In fact, it is known that miR-223 is an important pro-inflammatory factor in lung infiltration, since it mediates the inflammatory cascade response and consequent endothelial cell dysfunction, as well as disease progression [58,59,60,61].

Given the positive correlation between the expression levels of EV-hsa-miR-223-3p and those of EV-hsa-miR-24-3p and EV-hsa-miR-15b-5p, it was also interesting to investigate their shared gene targets, since the high expression of these EV-hsa-miRNAs may lead to a synergistic effect on their common targets. The transcription factor MAFB is one of the three common gene targets of miR-223-3p and miR-24-3p and plays a noteworthy role in the immune system, acting as a critical checkpoint in macrophages and influencing COVID-19 severity [62]. According to Vega et al., MAFB is related to defective type I IFN production, restrained antiviral response, and promotion of pro-fibrotic response [62]. This transcription factor suppresses CD8+ and CD4+ T cell activity and promotes the anti-inflammatory M2 macrophage polarization. It enhances the production of anti-inflammatory cytokines such as IL-10 and TGF-β while limiting pro-inflammatory cytokine expression [63]. Regarding other common targets of these miRNAs, Dupuis-Maurin et al. found that *Sp1* is also implicated in the activation of the immune system, with multiple functions, such as activation of antiviral response through the RIG-I pathway [64]. As a result, it can be hypothesized that the dysregulation of EV-hsa-miR-24-3p and EV-hsa-miR-223-3p may lead to a complex disruption of antiviral responses, impacting both anti-inflammatory and pro-inflammatory factors.

**Figure 7:**
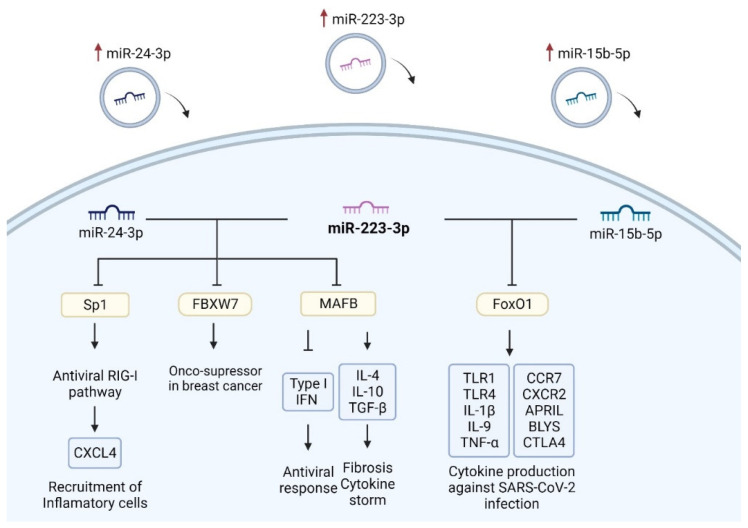
Signalling pathways of common target genes of elevated EV-hsa-miRNAs. The upregulation of miR-223-3p and miR-24-3p play a negative regulatory role in Sp1, leading to the subsequent inhibition of targets within the antiviral RIG-I pathways, such as CXCL4. This inhibition blocks the pathway’s ability to recruit inflammatory cells like neutrophils and macrophages. Additionally, both EV-hsa-miRNAs target the significant onco-suppressor in breast cancer, FBXW7, further affirming their dysregulation within the cancer context. Furthermore, inhibiting MAFB can disrupt signalling pathways associated with the response to SARS-CoV-2 infection, as this target serves as a crucial macrophage checkpoint in cytokine storm and antiviral response. The inhibition caused by the upregulation of miR-15b-5p and miR-223-3p on the FoxO1 transcription factor leads to a subsequent restriction of a potential therapeutic agent against SARS-CoV-2 infection, given its pivotal role in cytokine production, which is essential in combating the infection. Created with BioRender.

The common gene target of miR-15b-5p and miR-223-3p, *FoxO1*, is an important transcriptional factor that regulates vital cellular processes, including survival, apoptosis, oxidative stress response, and immune cell development [65]. The regulation of FoxO1 by mTOR and AKT plays a pivotal role in modulating the inflammatory response in DCs [66]. In fact, there is a study highlighting the significant therapeutic potential of FoxO in combating COVID-19, hypothesizing that activation of FoxO could represent a promising anti-inflammatory approach against SARS-CoV-2 infection [67]. If targeting FoxO1 is considered as a therapeutic approach for COVID-19, assessing hsa-miR-223-3p and hsa-miR-15b-5p levels becomes crucial. Inhibiting this transcription factor may impact the regulation of immune cells, making prior analysis essential to evaluate the effectiveness of the therapeutic approach against COVID-19 (Figure 7).

After discovering that these targets inhibit mRNA encoding the spike protein and also inhibit key immune signaling pathways, such as RIG-I via the *Sp1* gene and FoxO pathways through *FoxO1*, it can be concluded that the differing levels of these EV-hsa-miRNAs impact the effectiveness of the mRNA vaccine, suggesting their effect in cellular immunity, particularly in the production of cytokines and chemokines in the immune system against SARS-CoV-2 infection.

Overall, the results suggest that the post-vaccination elevation of specific EV-derived microRNAs, namely EV-hsa-miR-15b-5p, EV-hsa-miR-24-3p, and EV-hsa-miR-223-3p, may contribute to the modulation of signaling pathways within the immune system in response to COVID-19 vaccination. This is attributed to the ability of extracellular vesicles to transport these microRNAs to immune cells, leading to the dysregulation of cytokine and chemokine secretion, thereby influencing the host’s innate and adaptive immune responses. The interplay between their influence in suppressing the spike protein’s mRNA and other pathways of the immune system might highlight new perspectives on the outcomes of COVID-19 vaccination in cancer patients.

## 5. Conclusions

The development of safe and effective COVID-19 mRNA vaccines among cancer patients can be assisted by a better understanding of biomarker expression, allowing for predicting vaccine outcomes. EV-derived microRNAs have fundamental roles in regulating the expression and function of key immunological mediators. MiRNA expression profiles have been identified and are useful predictors for several inflammatory diseases. 

The elevated plasma levels of EV-hsa-miR-223-3p, EV-hsa-miR-24-3p, and EV-hsa-miR-15b-5p after COVID-19 booster dose could be attributed to COVID-19 vaccination. Moreover, it is worth noting that these EV-hsa-miRNAs could also be associated with specific immunological agents related to cellular immunity and could be potential biomarkers for vaccine efficacy in cancer patients. The elevated expression of EV-hsa-miR-223-3p levels could indirectly attenuate cellular immunity, since it has two gene targets, *Sp1* and *FoxO1*, that are recognized as promoters in antiviral response and considered potential therapeutic agents in SARS-CoV-2 infection, respectively. 

Further investigation is needed to confirm the roles and regulatory processes of these EV-microRNAs in the cancer patient’s immune modulation after COVID-19 vaccination. A comprehensive analysis of the association between these EV-hsa-miRNAs, the mRNA encoding the spike protein, and adaptative immune responses is needed to further validate the applicability of EV-miRNAs as biomarkers for monitoring the patient’s response to vaccination and, ultimately, stratify them according to their responses. 

## Figures and Tables

**Table 1:** Study of cohort’s population, with the number of subjects for each type of tumor, the sex, and the mean age for each group and number of patients with other comorbidities.

	Cohort Population	
*Number of patients*	*n*	%
Timepoint 1 (pre-boost)	54	100
Timepoint 2 (3 months post-boost)	50	92.59
Timepoint 3 (6 months post-boost)	40	74.07
*Mortality*		
Yes	9	16.67
No	45	83.33
*Tumor types*		
Breast	16	29.64
Lymphoma	8	14.82
Lung	7	12.96
Head and Neck	6	11.11
Rectal	6	11.11
Colon	4	7.41
Myeloma	2	3.70
Prostate	2	3.70
Bladder	1	1.85
Melanoma	1	1.85
Gastric	1	1.85
*Type of therapy*		
Chemotherapy	35	47.30
Targeted	12	16.22
Others	27	36.48
*Sex*		
Female	25	46.30
Male	29	53.70
*Age (Mean ± SD)*	58.85 ± 10.82	
*Boost vaccination brand*		
Pfizer-BioNTech	33	61.1
Moderna	16	29.63
Unknown	5	9.26

**Table 2:** Number of cancer patients with positive or negative anti-SARS-CoV-2 spike IgG levels in each timepoint.

	Positive Anti-SARS-CoV-2 Spike IgG Levels		Negative Anti-SARS-CoV-2 Spike IgG Levels	
	Solid tumors	Hematologic tumors	Solid tumors	Hematologic tumors
PreB (N = 54)	41	2	3	8
3 M (N = 50)	41	2	0	7
6 M (N = 40)	36	1	0	4

**Table 3:** Spearman correlation of EV-hsa-miRNAs profile and IgG anti-spike protein levels 3 months after boost vaccination.

	EV-hsa-miR-7-5p	EV-hsa-miR-15b-5p	EV-hsa-miR-24-3p	EV-hsa-miR-145-5p	EV-hsa-miR-223-3p
Spearman rho	0.085	−0.006	0.123	−0.075	0.088
*p*-value	0.624	0.970	0.394	0.607	0.545
N	36	50	50	50	50

**Table 4:** Spearman correlation of EV-hsa-miRNAs profile and IgG anti-spike protein levels 6 months after boost vaccination.

	EV-hsa-miR-7-5p	EV-hsa-miR-15b-5p	EV-hsa-miR-24-3p	EV-hsa-miR-145-5p	EV-hsa-miR-223-3p
Spearman rho	0.009	0.059	0.024	−0.144	0.151
*p*-value	0.965	0.717	0.881	0.376	0.351
N	25	40	40	40	40

## Data Availability

The data presented in this study are available on request from the corresponding author since these data are included in a larger ongoing project.
